# Blue Light Acts as a Double-Edged Sword in Regulating Sexual Development of *Hypocrea jecorina* (*Trichoderma reesei*)

**DOI:** 10.1371/journal.pone.0044969

**Published:** 2012-09-18

**Authors:** Chia-Ling Chen, Hsiao-Che Kuo, Shu-Yu Tung, Paul Wei-Che Hsu, Chih-Li Wang, Christian Seibel, Monika Schmoll, Ruey-Shyang Chen, Ting-Fang Wang

**Affiliations:** 1 Institute of Molecular Biology, Academia Sinica, Taipei, Taiwan; 2 Research Area Gene Technology and Applied Biochemistry, Institute of Chemical Engineering, Vienna University of Technology, Vienna, Austria; 3 Department of Biochemical Science and Technology, National Chiayi University, Chiayi, Taiwan; Seoul National University, Republic of Korea

## Abstract

The industrially important cellulolytic filamentous fungus *Trichoderma reesei* is the anamorph of the pantropical ascomycete *Hypocrea jecorina*. *H. jecorina* CBS999.97 strain undergoes a heterothallic reproductive cycle, and the mating yields fertilized perithecia imbedded in stromata. Asci in the perithecia contain 16 linearly arranged ascospores. Here, we investigated *H. jecorina* sexual development under different light regimes, and found that visible light was dispensable for sexual development (stroma formation and ascospore discharge). By contrast, constant illumination inhibited stroma formation, and an interruption of the darkness facilitated timely stroma formation in a 12 h/12 h light-dark photoperiod. The results of genetic analyses further revealed that *H. jecorina* blue-light photoreceptors (BLR1, BLR2) and the photoadaptation protein ENV1 were not essential for sexual development in general. BLR1, BLR2 and ENV1 are orthologues of the conserved *Neurospora crassa* WC-1, WC-2 and VVD, respectively. Moreover, BLR1 and BLR2 mediate both positive and negative light-dependent regulation on sexual development, whereas ENV1 is required for dampening the light-dependent inhibitory effect in response to changes in illumination. Comparative genome-wide microarray analysis demonstrated an overview of light-dependent gene expression versus sexual potency in CBS999.97 (MAT1–2) haploid cells. Constant illumination promotes abundant asexual conidiation and high levels of *hpp1* transcripts. *hpp1* encodes a h (hybrid)-type propheromone that exhibits features of both yeast a and a pheromone precursors. Deletion of *hpp1* could rescue stroma formation but not ascospore generation under constant illumination. We inferred that the HPP1-dependent pheromone signaling system might directly prevent stroma formation or simply disallow the haploid cells to acquire sexual potency due to abundant asexual conidiation upon constant illumination.

## Introduction

*Trichoderma* is a fungal genus present in nearly all soils as well as in other diverse habitats. *Trichoderma reesei* QM6a strain, originally isolated from tent canvas of the U.S. army in the Solomon Islands during World War II, and its derivatives have been applied to produce cellulolytic enzymes (cellulases and hemicellulases) and recombinant proteins for industrial uses. Recent molecular genetic studies indicate that *T. reesei* is an anamorph of the pantropical heterothallic ascomycete *Hypocrea jecorina* [1], [2]. A wild-type isolate, *H. jecorina* CBS999.97 strain, generates male and female haploids with either MAT1–1 or MAT1–2 mating-type locus, respectively. Hereafter, these two wild-type CBS999.97 haploids are denoted as CBS999.97(1–1) and CBS999.97(1–2). QM6a has a MAT1–2 mating type locus and can mate with CBS999.97(1–1) to form stromata (or fruiting bodies). Fertilized *H. jecorina* [CBS999.97(1–1) × CBS999.97(1–2) or CBS999.97(1–1) × QM6a] form stromata that contain asci with 16 ascospores [2]. *H. jecorina* has a normal a-factor-like peptide propheromone gene (*ppg1*) and a h (hybrid)-type a propheromone gene (*hpp1*) that exhibits features of both yeast *Saccharomyces cerevisiae* a and a pheromone precursors. Both *hpp1* and *ppg1* are transcribed during sexual development. However, it was reported that deletion of *hpp1* gene did not affect sexual development (mating, stroma formation, ascospore discharge) in daylight [3].

Light, one of the important environmental cues, affects all the living organisms on Earth directly or indirectly. For example, in *Neurospora crassa*, light plays a major role in its development such as the induction of carotenoid synthesis [4], promotion of conidiation [5], [6], direction of perithecial neck development [7] and entrainment of the circadian rhythm [8], [9]. Most light-induced phenotypes in *N. crassa* were reported to be dependent on two photoreceptor proteins, white-collar-1 (WC-1) and white-collar-2 (WC-2). The WC-1 protein functions as a blue-light receptor via its LOV (light, oxygen, or voltage) domain and by binding to a flavin adenine dinucleotide (FAD) chromophore. WC-1 also physically interacts with WC-2 to form the heteromultimeric “white-collar complex” (WCC) [10]. *N. crassa* also has a light adaptation protein, Vivid (VVD), which is another member of the LOV domain family and acts as a photoreceptor [11]. VVD performs its function by both ligand binding and protein-protein interaction. VVD is localized in the nucleus upon illumination [12] and mediates photoadaptation by interaction with WCC [13], [14], [15].

The blue-light response is an evolutionarily conserved signaling pathway present in almost all asco- and basidiomycetous taxa [14], [16], [17]. In *H. jecorina*, the orthologs of conserved WC-1, WC-2, and VVD are BLR1, BLR2, and ENVOY (ENV1), respectively [18], [19], [20], [21]. BLR1 and BLR2 mediate blue-light-induced mycelial growth and expression of cellulase genes. ENV1 functions in a negative regulatory feedback to tolerate continuous exposure to light and dampens the capacity of the fungus to perceive changes in light intensity [13]. This regulatory feedback involves functional interactions of ENV1 with the G-protein/cAMP/protein kinase A (PKA) signaling pathway. ENV1 can either influence cAMP levels via its action on the transcription levels of Ga subunits [22], [23] or dampen the outputs of Ga by its inhibitory effect on cAMP phosphodiesterase (PDE) [21].

As with other fungi, light is important for initiation of sexual development in *H. jecorina* [2], [24]. Given the scarcity of information on the mechanisms and environmental regulations of sexual development in *H. jecarina*, the objective of the present study was to explore whether visible light regulates initiation of sexual development by monitoring stroma formation. Our results suggest that BLR1, BLR2 and ENV1 play regulatory roles during these processes, and that predominate light-induced asexual conidiation may disallow sexual development.

## Results

### Sexual Development of *H. jecorina* is Sensitive to Light

**Figure 1:**
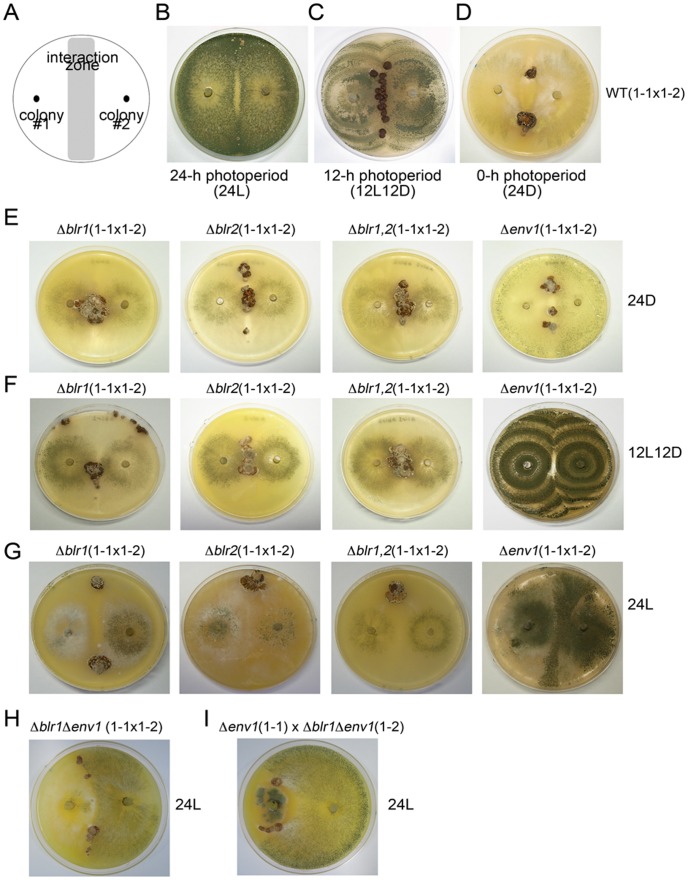
Blue-light perception and signaling regulate *H. jecorina* sexual development. (*A*) Schematic representation of the mating assay. Two single-ascospore cultures (#1 & #2) were inoculated on a 10-cm MEA plate as indicated. If sexual development occurs, stromata will be found at the interaction zone. (*B–D*) Sexual development of wild-type CBS999.97(1–1)×CBS999.97(1–2) strain under a 24L, 12L12D, or 24D photoperiod. (*E–G*) Sexual development of Δ*blr1*, Δ*blr2*, Δ*blr1* Δ*blr2* (Δ*blr1*,*2*) and Δ*env1* diploid mutants under a 24-h, 12-h, or 0-h photoperiod. (*H*) Sexual development of crossing Δ*blr1* Δ*env1* (1–1x1–2) under constant illumination. (*I*) Sexual development of crossing Δ*env1*(1–1) x Δ*blr1* Δ*env1*(1–2) under constant illumination.

**Figure 2:**
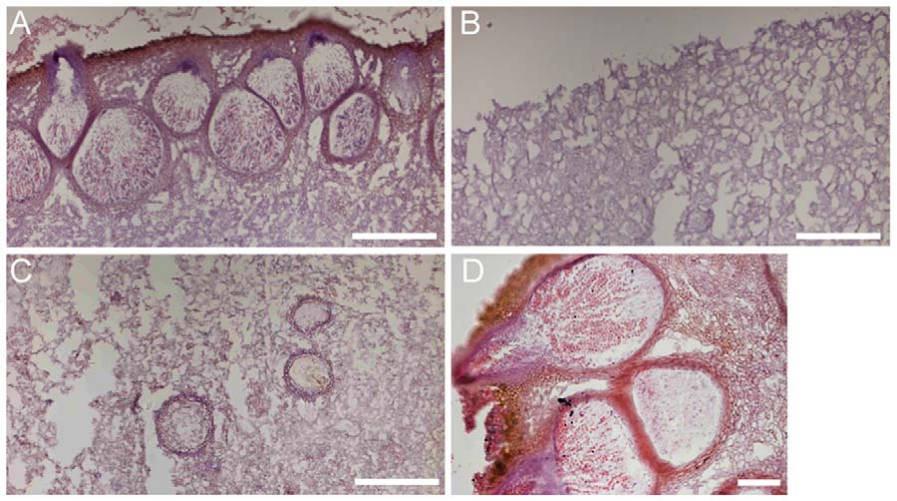
Light affects perithecia development. CBS999.97(1–1)×CBS999.97(1–2) crossing were carried out under a 12L12D photoperiod for 12 days (*A*) or in constant darkness for 7 days (*B*, *C*) or 14 days (*D*), respectively. Frozen sections of stromata were visualized by hematoxylin and eosin stain. (White scale bars: 50 mm).

To monitor sexual development, CBS999.97(1–1) and CBS999.97(1–2) were cultured on a malt extract agar (MEA) plate (see “Materials and Methods”). Stromata were expected to form at the interaction zone (Figure 1A). We found that stromata hardly developed under a 24 h photoperiod (24L) even after 30 days. Thus, constant illumination inhibits mating and stroma induction. By contrast, under a 12 h photoperiod (12L12D), stromata with dark brown pigmentation were observed at the interaction zone after 7–9 days. The diameter of stromata usually ranged from 2–5 mm (Figure 1C). Stroma formation occurred much more slowly under a 0-h photoperiod (24D; constant darkness) than under a 12L12D photoperiod, explaining why it was reported that light was required for stroma formation [2]. The stromata that developed under a 24D photoperiod had pale brown pigmentation, and a diameter of up to 1–2 cm (Figure 1D). Similar results were observed when CBS999.97(1–1) was crossed with QM6a under a 24L, 12L12D, or 24D photoperiod (data not shown). Haematoxylin and eosin staining for frozen sections of stromata revealed that perithecia were emerged and embedded into the upper surfaces of stromata generated under a 12L12D photoperiod (Figure 2A) [2]. In contrast, under a 24D photoperiod, perithecia developed more slowly and located deep inside the interior of stromata, and eventually having larger volumes and longer necks toward to the upper surfaces (Figure 2B–D). Together, our results indicate that different light regimes affect *H. jecorina* sexual development: constant illumination inhibited stroma formation, while unceasing darkness, compared to the 12L12D photoperiod, slowed down stroma formation. Therefore, an interruption of the darkness under a 12L12D photoperiod that mimics the natural photoperiod represented the optimal condition for sexual development.

### The Δ*blr1* and Δ*blr2* Mutants are Blind to Light during Sexual Development

*H. jecorina* BLR1 and BLR2 are required for induction of *env1* transcript in response to blue or white light during vegetative growth. It is also known that white or blue light slows down vegetative growth of the Δ*env1* mutant [19], [20], [21], [25]. Here we determined whether light influenced sexual development of Δ*blr1*, Δ*blr2*, Δ*blr1* Δ*blr2* (Δ*blr1,2*), and Δ*env1* mutants in the CBS999.97 background. Each of these four mutants, like the wild-type parental strains (Figure 1D), slowly developed large stromata under a 24D photoperiod (Figure 1E). Thus, BLR1, BLR2 and ENV1 were not essential for *H. jecorina* sexual development under constant darkness. Each of the Δ*blr1*, Δ*blr2*, and Δ*blr1,2* mutants also slowly formed large stromata under either a 12L12D photoperiod (Figure 1F) or a 24L photoperiod (Figure 1G), just as under a 24D photoperiod (Figure 1E). Therefore, BLR-dependent blue-light perception not only inhibited stroma formation under a 24L photoperiod (Figure 1B) but also accelerated stroma formation under a 12L12D photoperiod (Figure 1C). All stromata of wild-type and mutant strains described here generated dechexads with 16 ascospores (data not shown), indicating that BLR-dependent blue-light perception was also not essential for meiosis and ascospore formation.

### The Δe*nv1* Mutant cannot Respond to Changes in Light Illumination

The CBS999.97 Δ*env1* mutant did not form stroma under a 12L12D photoperiod (Figure 1F) or a 24L photoperiod (Figure 1G). On the other hand, wild-type CBS999.97 could form stromata under a 12L12D photoperiod but not under a 24L photoperiod (Figure 1C). Thus, Δ*env1* mutants failed to sense an interruption of darkness under a 12L12D photoperiod. We also showed that the Δ*env1* mutant could develop stromata and ascospores under a 24D photoperiod (Figure 1E, right panel). Since transcription of the *env1* gene is induced by light [19], [20], [21], the level of *env1* transcript in the wild-type CBS999.97 was very low under a 24D photoperiod (see blow). It is not surprising that loss of the *env1* gene does not affect sexual development under constant darkness.

To further confirmed if the function of ENV1 was dependent on illumination, we constructed the Δ*blr1* Δ*env1* double mutants. Fertilized Δ*blr1* Δ*env1* double mutant could form stromata under a 12L12D photoperiod (data not shown) or under a 24L photoperiod (Figure 1H). Similar results were observed when a Δ*env1* single mutant was crossed with a Δ*blr1* Δ*env1* double mutant under a 12L12D photoperiod (data not shown) or under a 24L photoperiod (Figure 1I). Because the Δ*env1* mutants could not form stroma under a 12L12D (Figure 1F) or 24L photoperiod (Figure 1G), we inferred that the Δ*env1* mutants might display a light-dependent defect in either female fertility or male fertility.

### The Conidia of CBS999.97 Δ*env1* are Functional Spermatia

**Figure 3:**
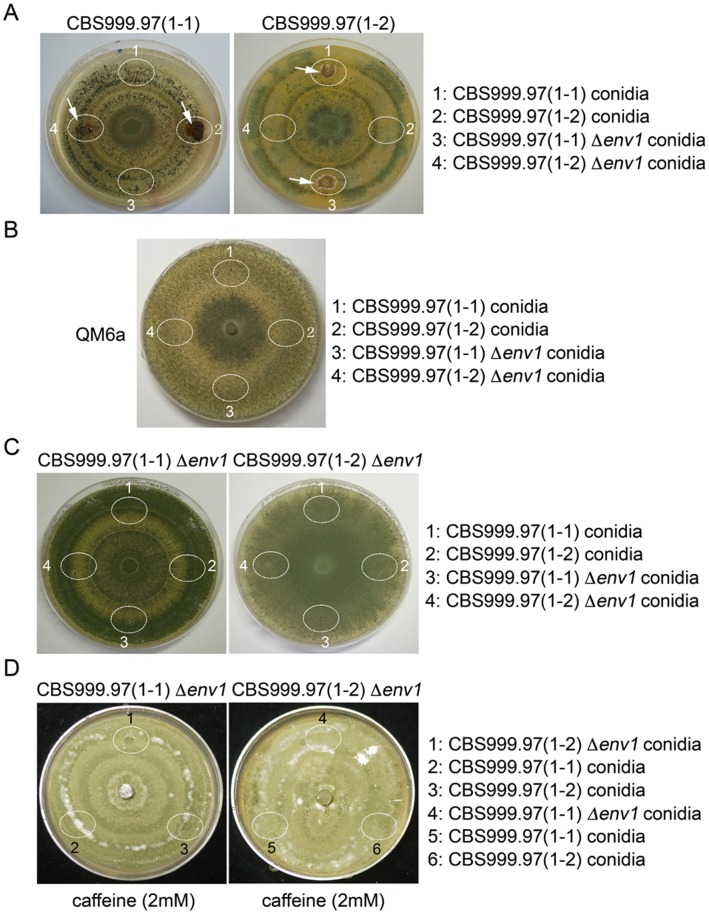
Stroma induction by conidia in a 12L12D photoperiod. Female recipient strains grew on a 10-cm MEA plate under a 12L12D photoperiod for 7 days, including CBS999.97(1–1), CBS999.97(1–2) (*A*), QM6a (*B*), and CBS999.97(1–1) Δ*env1*, and CBS999.97(1–2) Δ*env1* mutants (*C*, *D*). The conidia from male strains were spotted onto an indicated white oval region of the female recipient MEA plate. The MEA plate was incubated under a 12L12D photoperiod for 10–14 days. Caffeine (2 mM) was added to the MEA plate in (*D*). The stromata were marked as indicated.

**Figure 4:**
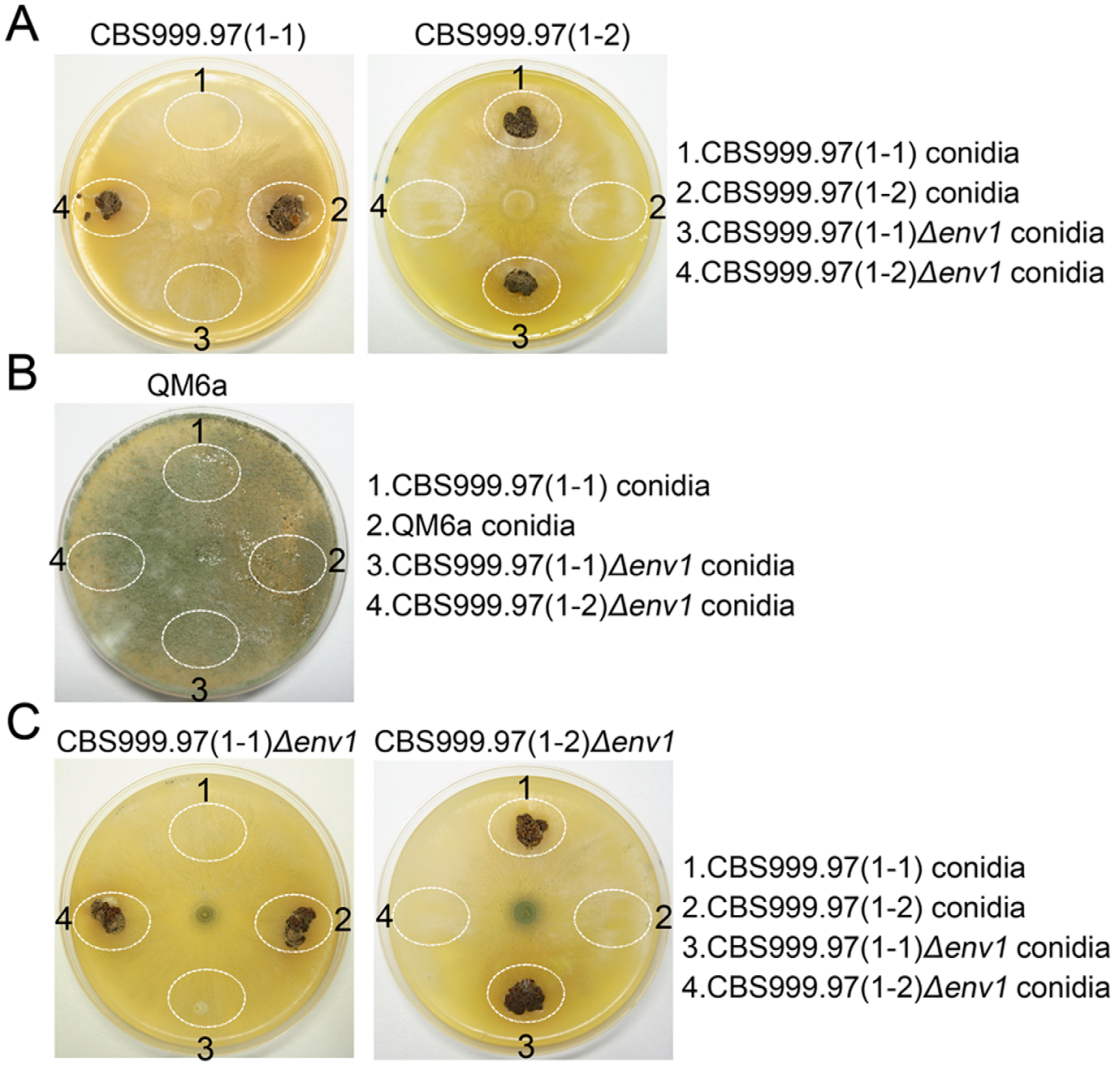
Stroma induction by conidia in constant darkness. Female recipient strains grew on a 10-cm MEA plate under a 12L12D photoperiod for 7 days, including CBS999.97(1–1), CBS999.97(1–2) (*A*), QM6a (*B*), and CBS999.97(1–1) Δ*env1*, and CBS999.97(1–2) Δ*env1* mutants (*C*). The conidia from a male strain were spotted onto an indicated white oval region of a female recipient MEA plate. The MEA plate was incubated under a 24D photoperiod (constant darkness) for 10–14 days. The stromata were marked as indicated.

Sexual crosses could be performed by mixing the conidia from a CBS999.97 haploid male strain to the mycelia of a CBS999.97 haploid female strain (Figure 3A). Next, we determined if ENV1 is required for male or female fertility. In brief, the conidia of a male strain were applied to a MEA plate where a female strain has grown into mycelia, and incubated for additional 14 days under a 12L12D photoperiod (Figure 3) or under a 24D photoperiod (Figure 4). As shown in Figures 3A and 4A, both CBS999.97(1–1) and CBS999.97(1–1) Δ*env1* conidia induced a CBS999.97(1–2) recipient female strain to form stroma structures, whereas CBS999.97(1–2) and CBS999.97(1–2) Δ*env1* conidia also induced the CBS999.97(1–1) recipient female strain to form stroma structures. In contrast, conidia from donor strains did not induce stroma formation in female recipient strains with the same mating type. These results indicated that the Δ*env1* mutant, like wild-type strain, was male fertile under a 12L12D photoperiod. Thus, *env1* deletion or blue-light illumination apparently does not affect male fertility.

QM6a is female sterile [2]. Using the female fertility assay described here, we confirmed that neither CBS999.97(1–1) conidia nor CBS999.97(1–1) Δ*env1* conidia induced QM6a to develop any stroma under a 12L12D photoperiod (Figure 3B) or in 24D photoperiod (Figure 4B). These results also indicated that the method we used for conidia isolation was able to exclude mycelia, because QM6a could mate with CBS999.97(1–1) mycelia to form stromata and ascospores using the crossing method described in Figure 1A (data not shown) [2].

### The *Δenv1* Mutants become Female Sterile in Response to Light

Intriguingly, we found that CBS999.97(1–1) Δ*env1* and CBS999.97(1–2) Δ*env1* were female sterile under a 12L12D photoperiod (Figure 3C) or a 24L photoperiod (data not shown). By contrast, they were not female sterile under a 24D photoperiod (Figure 4C; also see Figure 1E, right panel). As the Δ*env1* mutants showed no defect in male fertility, our result indicated that the lack of *env1* leads to perturbation of light-mediated regulatory mechanism responsible for stroma formation.

Caffeine, a phosphodiesterase (PDE) inhibitor, alleviated the growth defect of Δ*env1* mutants in response to light, which was interpreted as ENV1 having an inhibitory effect on PDE [21]. However, we found that caffeine did not rescue the female sterility of CBS999.97(1–1) Δ*env1* or CBS999.97(1–2) Δ*env1* under a 12-h photoperiod (Figure 3D).

From these findings, we conclude that BLR proteins receive light and respond by inhibiting stroma formation with constant illumination (24L), but not under a 12L12D photoperiod, and that the photoadaptation protein ENV1 desensitizes this inhibitory effect under a 12L12D photoperiod. This BLR-mediated inhibition specifically affects female fertility. In addition, the effect of ENV1 on stroma development is regulated by an unknown pathway that is apparently independent of the cAMP signaling pathway.

### Genome-wide Analysis of Light-dependent Transcription in *H. jecorina*

Because our results indicate that blue light acts as a double-edged sword in regulating *H. jecorina* sexual development, we were interested in gaining an overview on the light-dependent regulatory mechanisms.

**Figure 5:**
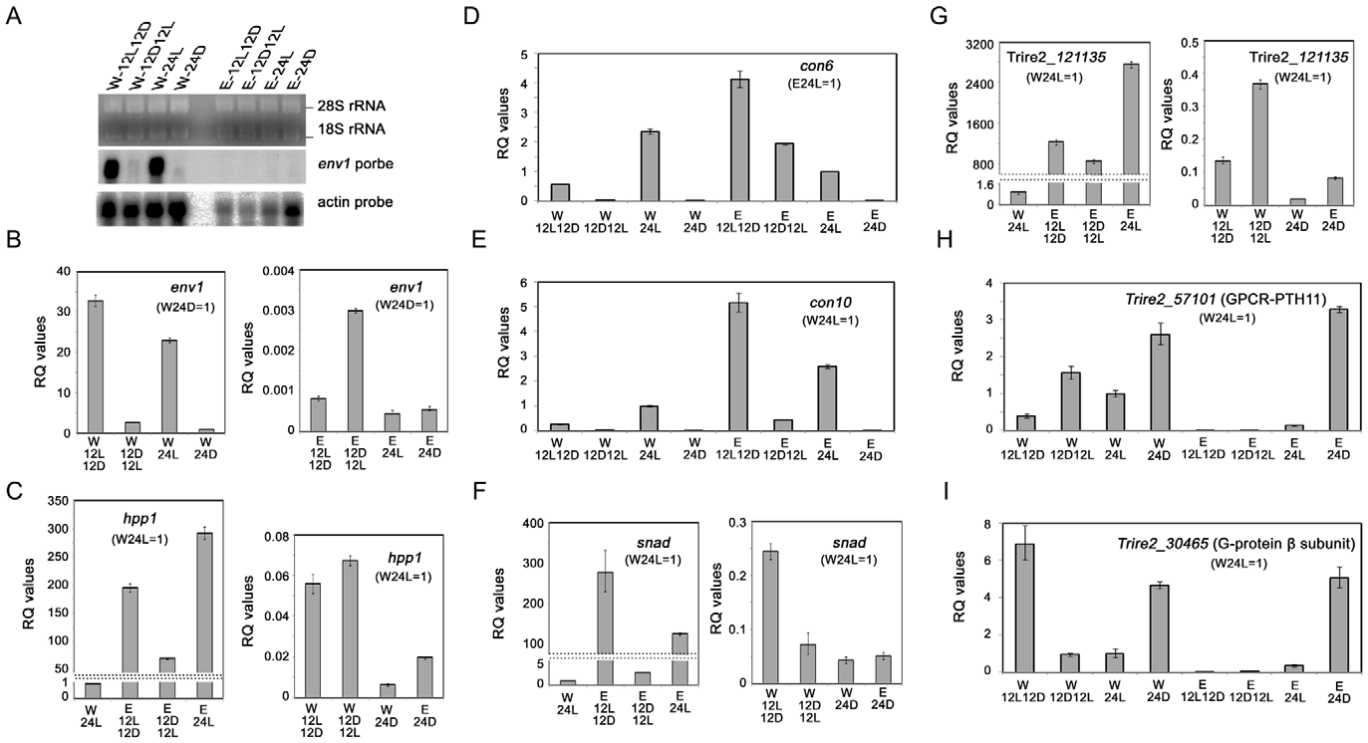
Transcription levels of putative genes involved in conidiation and sexual potency. Total RNAs were extracted from 8 different experimental conditions as indicated. The quality of extracted RNA samples was further analyzed with the RNA 6000 Nano kit by Agilent 2100 Bioanalyzer (see Materials and Methods). (*A*) Northern blots analysis of *act1* (actin) and *env1* transcription. The denaturing RNA agarose gel was stained with ethidium bromide, the 18S rRNA and 28S rRNA bands were clearly visible in the intact RNA samples. (*B–H*) qRT-PCR. Relative transcript abundance of representative genes in sexually potent and impotent conditions. Data were given as relative quantitative (RQ) values to one of the eight conditions as indicated. The transcripts of the ribosome protein gene *rpl6e* were used for normalization of the qRT-PCR data [43].

*H. jecorina* [CBS999.97(1–2) or CBS999.97(1–2) Δ*env1*] was grown in four different light/dark conditions, and there was no mating partner on the plates. Genome-wide transcriptional analysis using microarrays was applied (Gene Expression Omnibus accession number GSE39111) to identify alternations in gene regulation in sexually potent and impotent conditions. The four sexually potent conditions examined here were: (I) W-24D: CBS999.97(1–2) in constant darkness for 7.25 days; (II) W-12L12D: CBS999.97(1–2) in a 12 h light/dark cycle for 7 days then with additional 6 h light illumination; (III) W-12D12L: CBS999.97(1–2) in a 12 h dark/light cycle for 7 days and then with additional 6 h in darkness; (IV) E-24D: CBS999.97(1–2) Δ*env1* in constant darkness for 7.25 days. By contrast, the four sexually impotent conditions are: (I) W-24L: CBS999.97(1–2) with constant illumination for 7.25 days; (II) E-12L12D: CBS999.97(1–2) Δ*env1* in a 12 h light/dark cycle for 7 days and then with additional 6 h light illumination; (III) E-12D12L: CBS999.97(1–2) Δ*env1* in a 12 h dark/light cycle for 7 days and then with additional 6 h constant darkness; (IV) E-24L: CBS999.97(1–2) Δ*env1* with constant illumination for 7.25 days. As internal controls, we first checked the *env1* gene expression in these eight conditions by Northern blot hybridization (Figure 5A) and qRT-PCR (Figure 5B). Transcription of *env1* was higher with illumination (e.g., W-24L and W-12L12D) than in darkness (e.g., W-12D12L and W-24DD). These results correlated with earlier data that transcription of *env1* gene was dependent on light illumination [19], [26]. The results of qRT-PCR further indicated that, with the level of *env1* transcripts in W-24D set to 1, only background levels (<0.003) of *env1* transcript in the four CBS999.97(1–2) Δ*env1* conditions (E-24L, E-12L12D, E-12D12L and E-24D) (Figure 5B). Hence, we confirm that growth conditions and microarray methods were appropriate for our analysis.

**Figure 6:**
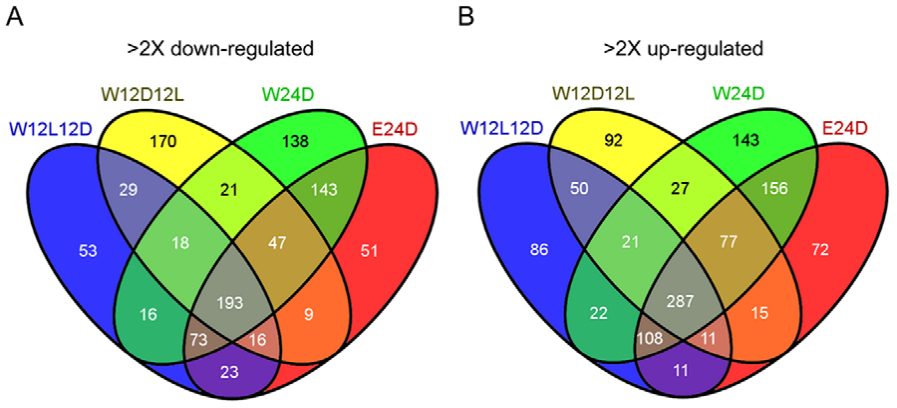
Regulatory targets of sexual potency in the CBS999.97(1–2) wild-type strain (W) and the CBS999.97(1–2) Δ*env1* mutant (E). VENN diagram of genes 2-fold downregulated genes (*A*) and 2-fold up-regulated genes (*B*) in the four sexually potent conditions (W-24D, W-12L12D, W-12D12L, E24D) in comparison with the four sexually impotent conditions (E24L, E-12L12D, E-12D12L, W-24L). For details on gene regulation see supplementary files (Tables S1 and S2).

*H. jecorina* wild-type strain and the Δ*env1* mutant show considerable alternations in gene regulation in dependence on their sexual potency. For example, we found that 193 genes were at least two-fold down-regulated under 4 sexually potent conditions (i.e., W-12L12D, W-12D12L, W-24D and E-24D) in comparison with those under the four sexually impotent conditions (i.e., E-12D12L, E-12L12D, E-24L and W-24L) (Figure 6A; Table S1). Among these down-regulated genes, we found several evolutionarily conserved genes, including 3 conidiation-specific genes (*con-6*, *con-10*, *sand*), a novel gene (Protein Id: TRIRE2_121135), a homolog of yeast *AQY1* water channel gene, two DNA repair enzyme photolyase genes (e.g., *phr1*), the *mata1* mating type gene, the h-type a propheromone gene (*hpp1*), and several genes involved in a propheromone processing and secretion (e.g., *ste6*, *ste14*, *ste24*, *ram2*, *sir2*) (Tables 1 and 2). *con-6* and *con-10* are two conidiation genes that were identified based on their preferential expression during *N. crassa* macroconidiophore development [27], [28]. Both *con-6* and *con-10* showed a heighten response to photoinduction in the *N. crassa Dvvd* mutant [29]. *N. crassa* VVD is the orthologue of *H. jecorina* ENV1. The *sand* gene encodes a spindle pole body-associated protein that affects septation and conidiation in *Apsergillus nidulans* [30]. Yeast AQY1 gene encodes a spore-specific water channel that mediates the transport of water across cell membrane, and the Aqy1 protein was reported to be involved in spore maturation and freeze tolerance by allowing water outflow [31]. In *T. harzianum* and *T. atroviride, phr1* gene transcripts were induced in conidia during development and spore formation [32], [33]. *hpp1* encodes *H. jecorina* h-type a propheromone protein that is not essential for sexual development in daylight [3]. Transcriptional co-induction of *hpp1* with several conidiation-specific genes in the four sexually impotent conditions raises an intriguing possibility that *hpp1* may have a role in promoting conidiation under constant illumination (see below). Alternatively, the pheromone system might get completely out of balance in the Δ*env1* mutants upon illumination and thus the mycelia suffered a loss of sexual identity [25].

**Table 1:** Annotation of 15 representative genes that are transcriptionally down-regulated in the four sexually potent conditions.

Gene I.D.	Annotation
Trire2_34312	*Neurospora crassa* conidiation-specific gene 6 (con-6) [Genbank: P34762].
Trire2_5084	*Neurospora crassa* conidiation-specific gene 10 (con-10) [Genbank: AAA33572].
Trire2_107856	A homolog of spindle pole body-associated protein (SNAD), affects septation and conidiation in Aspergillus nidulans [Genbank: AF070480].
Trire2_121135	A novel gene.
Trire2_82321	*Saccharomyces cerevisiae* AQY1, an spore-specific water channel gene that mediates the transport of water across cell membranes, developmentally controlled; may play a role in spore maturation.
Trire2_64667	*Neurospora crassa* glucose-repressible gene (*grg-1*) [Genbank: CAC28672].
Trire2_107680	Class I DNA photolyase Phr1, rapidly regulated by blue light in *Trichoderma Harzianum*.
Trire2_77473	hypothetical class 1 DNA photolyase.
Trire2_124341	*mata1* encodes mating type protein MATa1, contains HMG-box.
Trire2_34493	*hpp1* (h-type propheromone).
Trire2_31134	*Saccharomyces cerevisiae* Ste14 farnesyl cysteine-carboxyl methyltransferase, mediates the carboxyl methylation step during C-terminal CAAX motif processing of a-factor and RAS proteins in the endoplasmic reticulum.
Trire2_62693	Saccha*romyces cerevisiae* Ste6, a plasma membrane ATP-binding cassette (ABC) transporter required for the export of a-factor
Trire2_124222	Sa*ccharomyces cerevisiae* Ste24, highly conserved zinc metalloprotease that functions in two steps of a-factor maturation, C-terminal CAAX proteolysis and the first step of N-terminal proteolytic processing.
Trire2_22093	*Saccharomyces cerevisiae* Ram2, the alpha subunit of both the farnesyltransferase and type I geranyl-geranyltransferase that catalyze prenylation of proteins containing a CAAX consensus motif; essential protein required for membrane localization of Ras proteins and a-factor.
Trire2_67057	*Saccharomyces cerevisiae* Sir2, a NAD+ dependent histone deacetylase of the Sirtuin family involved in regulation of lifespan; plays roles in silencing at HML, HMR, telomeres, and the rDNA locus; negatively regulates initiation of DNA replication. Sir2 interacts with two silencing and proteins, Sir3 (Ste8) and Sir4 (Ste9), which are required for production of mating pheromone.

**Table 2:** Relative transcriptional levels of 15 representative down-regulated genes in the 4 sexually potent conditions v.s. the 4 sexually impotent condition.

	W-12D12L	W-12L12D	W-24D	E-24D
Protein ID (gene name)	Folds reduced (p value)	Folds reduced (p value)	Folds reduced (p value)	Folds reduced (p value)
Trire2_34312 (*con6*)	19.31 (0.000017)	4.28 (0.004512)	42.27 (0.000002)	40.36 (0.000002)
Trire2_5084 (*con10*)	3.29 (0.000804)	2.48 (0.008011)	69.52 (<0.000001)	31.72 (<0.000001)
Trire2_10785 (*snad*)	64.58 (0.000897)	50.69 (0.001372)	66.78 (0.000838)	69.04 (0.000793)
Trire2_121135	45.23 (<0.000001)	100.75 (<0.000001)	140.09 (<0.000001)	125.99 (<0.000001)
Trire2_82321 (*aqy1*)	16.58 (0.000013)	15.91 (0.000013)	27.6 (0.000002)	25.2 (0.000003)
Trire2_64667 (*grg1*)	17.44 (<0.000001)	4.93 (0.000079)	45.33 (<0.000001)	36.9 (<0.000001)
Trire2_10768 (*phr1*)	20.12 (0.000009)	3.04 (0.001317)	23.48 (<0.000001)	69.04 (0.000793)
Trire2_77473 (*phr1 like*)	7.96 (0.000975)	5.47 (0.002419)	28.07 (0.000012)	23.15 (0.000018)
Trire2_12434 (*mata1*)	5.33 (0.000191)	3.59 (0.000387)	5.53 (0.000033)	5.98 (0.000018)
Trire2_34493 (*hpp1*)	37.25 (<0.000001)	63.99 (<0.000001)	150.15 (<0.000001)	117.95 (<0.000001)
Trire2_31134 (*ste14*)	4.47 (0.000097)	6.53 (0.000006)	8.65 (<0.000001)	8.58 (<0.000001)
Trire2_62693 (ste6)	4.16 (0.000158)	5.51 (0.000011)	6.62 (0.000006)	6.67 (0.000005)
Trire2_12422 (*ste24*)	4.87 (0.000019)	4.48 (0.000019)	4.04 (0.000028)	5.01 (0.000012)
Trire2_22093 (*ram2*)	6.5 (0.000051)	4.39 (0.000131)	4.32 (0.000069)	4.75 (0.000055)
Trire2_67057 (*sir2*)	4.2 (0.000075)	2.89 (0.000432)	3.53 (0.000071)	3.15 (0.000202)

The results of qRT-PCR (Figure 5B–F) experiments further confirmed that microarray results could reflect actual expression patterns in the samples. Exceptionally high-levels of *hpp1* (Figure 5C), *con-6* (Figure 5D), *con-10* (Figure 5E), *sand* (Figure 5F) and *121135* (Figure 5G) transcripts were observed in three sexually impotent conditions (i.e., E-12D12L, E-12L12D, E-24L). The transcription levels of these five genes were also higher in CBS999.97(1–2) with constant illumination (W-24L) than in all four sexually potent conditions (W-12L12D, W-12D12L, W-24D, E24D). These results suggest that wild-type CBS999.97(1–2) and Δ*env1* mutant apparently undergo abundant photoconidiation in the four sexually impotent conditions. This supposition is consistent with earlier reports that *Trichoderma* species produce conidia in response to blue light illumination [34]. Because ENV1 dampens the light-dependent effect in response to changes in illumination, the Δ*env1* mutant undergoes abundant photoconidiation even under a 12h light/dark cycle (i.e., E-12D12L and E-12L12D).

Our genome-wide transcription analyses also revealed that 287 genes are at least two-fold up-regulated under 4 sexually potent conditions (i.e., W-12L12D, W-12D12L, W-24D and E-24D) in comparison with those under the three sexually impotent conditions (i.e., E-12D12L, E-12L12D, E-24D) (Figure 6B; Table S2). Among these up-regulated genes, we found significant enrichment of genes involved in cellulase and hemicellulase metobolism (hydrolyases, cellulose binding proteins and transporters), electron transport, redox regulation, protein folding, etc. Because these metabolic genes are needed for vegetative growth, we infer that CBS999.97(1–2) and CBS999.97(1–2) Δ*env1* preferentially undergo hyphal growth (rather than conidiation) in these four sexually potent conditions. We also found several evolutionarily conserved genes are up-regulated, including a homolog of mold-specific M46 gene in a dimorphic fungus *Histoplasma capsulatum* (Genbank: AAL12252), a G protein β subunit gene, 3 hypothetical G protein coupled receptor (GPCR) genes, a myosin heavy chain gene, and 4 fungus-specific transcriptional factor genes (Table 3). The results of qRT-PCR experiments indicated that the G_β_ gene (Genbank: EGR45759) (Figure 5G) and the PTH11-type GPCR [Genbank: ERG51469] (Figure 5F) were transcribed higher in the four sexually potent conditions (W-12L12D, W-12D12L, W-24D, E-24D) or when the wild-type strain was under constant illumination (i.e., W-24L). Further work will be needed to examine if these genes are indispensable for sexual development.

**Table 3:** Annotation of representative genes that are transcriptionally up-regulated in the four sexually potent conditions.

Gene group or gene I.D.	Annotation
Group I	>30 Glycoside hydrolases or cellulose binding proteins (e.g., *cel5a*, *man5a*, *cle12a*, *cel61b*, *cel3a*, *cip2*, *cle7b*, *cip1*, etc)
Group II	>11 putative peptidases or proteinases
Group III	>25 genes involved in metabolic processes, including electron transport, oxidation-reduction reactions, etc.
Group IV	>16 major facilitator superfamily, sugar transporters or amino acid permeases
Trire2_122160	Heat shock protein DnaJ
Trire2_65819	Heat shock protein Hsp70
Trire2_104390	Glutathione S-transferase
Trire2_103135	Mold-specific M46 protein in a dimorphic fungus *Histoplasma capsulatum* [Genbank: AAL12252]
Trire2_30465	G protein β-subunit [Genbank: EGR45759]
Trire2_57101	Hypothetical PTH11-type GPCR [Genbank: ERG51469]
Trire2_53238	Hypothetical secretin-like GPCR [Genbank: ERG52173]
Trire2_66751	Hypothetical rhodopsin-like GPCR [Genbank: ERG46177]
Trire2_108143	Myosin, heavy chain
Trire2_110790	Fungal transcriptional regulatory proteins
Trire2_102497	Fungal transcriptional regulatory proteins
Trire2_122499	Fungal transcriptional regulatory proteins
Trire2_59740	Fungal transcriptional regulatory proteins

In summary, the results of genome-wide transcription analysis indicate that CBS999.97(1–2) or CBS999.97(1–2) Δ*env1* apparently elicit abundant photoconidiation in the four sexually impotent conditions. By contrast, these two strains undergo vegetative growth in the four sexually potent conditions. Constant illumination apparently induces photoconidiation in *H. jecorina,* and abundant conidiation may reduce its potency for sexual development.

### Roles of *hpp1* in *H. jecorina* Sexual Development Upon Constant Illumination

**Figure 7:**
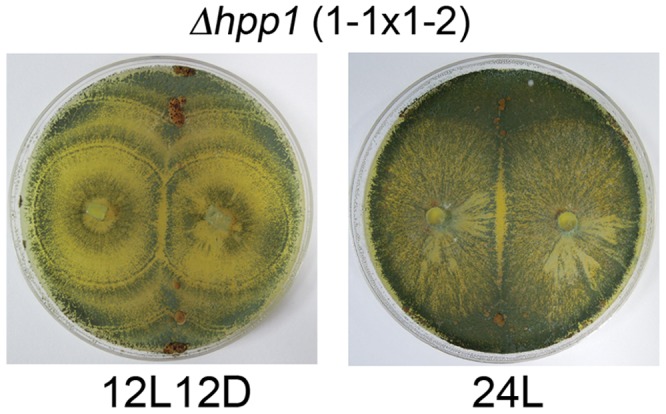
The effects of Δ*hpp1* deletion on stroma induction under a 12L12D or 24L photoperiod. Sexual development of the Δ*hpp1* mutant was determined as described in Figure 1.

It was reported that deletion of *hpp1* did not perturb CBS999.97 sexual development (stromata formation and ascospores discharge) in daylight [3]. We observed similar results when CBS999.97(1–1) Δ*hpp1* was crossed with CBS999.97(1–2) Δ*hpp1* under a 12L12D photoperiod (Figure 7, right panel). Because the wild-type CBS999.97(1–2) strain was sexually impotent and expressed a high level of *hpp1* transcripts under a 24L photoperiod (Figure 5), we then examined whether *hpp1* is responsible for infertility upon constant illumination. We found that deletion of *hpp1* could rescue stroma formation in a 24L photoperiod (Figure 7, left panel), however these stromata were smaller in size (diameter ∼1–2 mm) and contained no ascospores after 14 days (data not shown). Thus, overexpression of *hpp1* transcripts upon constant illumination is responsible for suppression of sexual mating or stroma formation.

## Discussion

Blue light regulates many physiological and developmental processes in fungi [35], [36]. *N. crassa* is the paradigm for studying the molecular mechanisms of blue-light responses. The two White Collar proteins (WC-1 and WC-2) and the photoadaptation protein VVD virtually mediate almost all light-induced phenotypes [15]. Upon the light induction, WC-1 and WC-2 form a WCC complex that binds to the promoters of light-responsive genes to trigger the transcription. Moreover, VVD mediate photoadaptation in which the phosphorylation of WC-1 results in the exclusion of WCC complex from the promoters of light-responsive genes [10]. Intriguingly, recent molecular studies in various *Trichoderma/Hypocrea* species have revealed conserved mechanisms of blue-light perception in mycelia growth and conidiation through the White Collar orthologs BLR-1/BLR2 and the VVD ortholog ENV1 [13], [20], [21], [33], [37] and other fungi [16]. Both BLR proteins and ENV1 are required for conidiation. In the present of light, ENV1 positively regulates the growth rate of mycelium. Gene expression analysis indicates that ENV1 expression depends on light, BLR1 and BLR2. ENV1 is essential for photoadaptation, and is able to alter the transcription pattern of light-dependent and light-independent genes [20], [26]. Herein, we demonstrated the effects of the three light sensing components on sexual development of *H. jecorina*. We provided evidences that light, BLR proteins and ENV1 were not essential for wild-type *H. jecorina* to induce sexual development. In addition, with the light exposure, BLR-mediated blue-light responses act as a double-edged sword for *H. jecorina* sexual development. The stroma development is blocked by constant illumination through the action of BLR proteins, but the perithecium formation is promoted under a 12L12D photoperiod compared with that in unceasing darkness. It is unknown how light facilitates perithecium formation, but it suggests that light may have a positive role in sexual development after stroma induction.

Compared with other reports regarding the effects of WC protein homologs on fungal sexual development, WC-1 and WC-2 of the basidiomycetes *Cryptococcus neoformans*, similar to our results, negatively regulate the sexual filamentation [38]. In contrast, *N. crassa* WC proteins induce the formation of protoperithecia [39]. *Aspergullus nidulans* LreA (WC-1) and LreB (WC-2) protein complex acts as an activator of sexual development, and is able to interact with FphA, a phytochrome for red light sensing. Intriguingly, the activities of LreA and LreB are repressed by light through the activity of FphA [40]. Thus, although WC proteins are evolutionally conserved among fungal species, their activities in regulating the sexual development may vary to adapt individual environmental niche.

*H. jecorina* ENV1 apparently has similar function as VVD in *N. crassa* [29], [41]. Combined with the effects of Δ*env1* or Δ*blr1* Δ*env1* under different light regimes (Figures 1), it appears that ENV1 is not directly involved in stroma development, but does play important roles in promoting sexual development and/or in inhibiting asexual conidiation by desensitizing BLR-mediated signaling responses. Based on the genome-wide transcription analyses described above, it is tempting to propose that *H. jecorina* BLR-mediated pathways induce both high levels of HPP1 pheromone and abundant asexual conidiation in CBS999.97(1–2) wild-type and Δ*env1* haploid strains under constant illumination. Further studies will be conducted to determine if and how the HPP1-dependent pheromone signaling system inhibits stroma formation upon constant illumination. Alternatively, HPP1 may promote abundant asexual conidiation and then prohibit the haploid cells to acquire sexual potency. Light also induces a high level of a-factor-like peptide pheromone PPG1 in CBS999.97(1–1) wild-type and Δ*env1* haploid strains [25], it is also of interest to examine the roles of PPG1 in sexual development under different light regimes.

Since the ENV1 homologs only present in ascomycetes, the interplay between BLR protein and ENV1 of *H. jecorina* may provide novel regulatory mechanisms on conidiation and sexual development in response to light. Consistently, our data further indicate that *H. jecorina* blue-light signaling machinery act via different mechanisms in regulating mycelial growth, asexual conidiation and sexual development. The blue light mediated mycelial growth in *H. jecorina* has been implicated by regulating the levels of cAMP through the inhibition of ENV1 on PDE [21], [22]. Addition of caffeine, a PDE inhibitor, rescued the growth defect of Δ*env1*, but did not restore the female fertility of Δ*env1*. This indicated that ENV1-mediated stroma formation is through a distinct pathway, rather than cAMP signaling pathway. A deeper insight into the blue-light signaling networks in *H. jecorina* sexual development undoubtedly will allow us to understand how fungi succeed in their natural habitats, as well as provide new knowledge for optimizing their industrial applications.

## Materials and Methods

### Microbial Strains, Culture Conditions, Mutant Construction and Cytology

Maintenance, single spore isolation, and stock culture of QM6a and CBS999.97 strains were performed as described previously [2]. To monitor sexual development, CBS999.97(1–1) was crossed with CBS999.97(1–2) or QM6a on a 10-cm malt extract agar (MEA) plate as previously described [2]. The MEA plate was incubated in a growth chamber at 25°C under a 24L, 12L12D, or 24D photoperiod, respectively. A plant growth chamber with a light (either white light intensity of about 80 µmol/m^2^/s or blue light with the wavelength of 440–460 nm) was used in this study.

Construction of Δ*blr1*, Δ*blr2*, Δ*blr1* Δ*blr2* (Δ*blr1*,*2*), Δ*env1*, and Δ*hpp1* mutants in CBS999.97(1–1) and CBS999.97(1–2) strains described previously [3], [25]. These deletion strains were confirmed by Southern blotting analysis using the DNA fragments of *blr1*, *blr2*, and *env1* genes, respectively (data not shown). Frozen sections of stromata were stained hematoxylin and eosin for visualization [42]. The Δ*blr1* Δ*env1* double mutant was obtained by crossing Δ*blr1* and Δ*env1* single mutants, and confirmed by genomic PCR for integration of the deletion hygromycine resistance cassettes and for removal of the wild-type gene (data not shown).

### Fertility Assay

To test male or female fertility, sexual crosses were performed using conidia from the male tester strains as spermatia or male gamates. In brief, haploid male parent strain was inoculated on the center a 6-cm MEA plate and incubated at 25°C in a plant growth chamber under a 12L12D photoperiod for 5–7 days. To collect conidia, 2 mL double distilled water was added on the pheromone donor MEA plate for 2 h. This solution was transferred to a 2-mL microcentrifuge tube, centrifuged at 3,300 × *g* for 6 min, and the top 500 mL of supernatant was harvested. 20 mL of supernatant were spotted onto a 10-cm MEA plate that had been inoculated with the haploid female parent strain. The MEA plate was then incubated at 25°C in a plant growth chamber under a 12L12D photoperiod (Figure 3) or a 24D photoperiod (Figure 4) for 14 days.

### Quantification of Gene Expression by Northern Blot and qRT-PCR

CBS999.97(1–2) or CBS999.97(1–2) Δ*env1* was cultured on a MEA plate covered by cellulose based cellophane sheet at 25°C. Only the mycelia that had not reached the edges of the plate were harvested and frozen by liquid nitrogen. The total RNA was extracted with the TRI Reagent Solution (Ambion, Carlsbad, CA) and the RNeasy Plant Mini Kit (Qiagen, Valencia, CA). Briefly, frozen mycelia were ground with a mortar and a pestle. Mycelial powder (100 mg) was mixed with one ml of TRI Reagent solution. Supernatant was centrifuged with 12000×*g* and transferred to a new microcentrifuge tube, and then mixed with 0.2 ml chloroform. After centrifugation, aqueous phase was carefully transferred to a new microcentrifuge tube and mixed with equal volume of 100% ethanol to precipitate RNA. The solution containing the precipitations was loaded onto the RNeasy spin column. Subsequent procedures followed the manufacture’s protocol, including on-column DNase digestion with the RNase-Free DNase set (Qiagen, Valencia, CA). The quality of extracted RNA was further analyzed with the RNA 6000 Nano kit by Agilent 2100 Bioanalyzer (Agilent Technologies, Palo Alto, CA). For Northern blot analysis, DNA probes were synthesized by using a Nick Translation kit (GE Healthcare, United Kingdom). The total RNA samples were electrophoresed, transferred to Gene Screen Plus membrane, and autoradiographed.

For qRT-PCR, total RNAs were converted into cDNAs using the oligo(dT)_20_ primers and the SuperScript III Fist-Strand Synthesis System (Invitrogen, Carlsbad, CA). Gene-specific primers (Table S3) were designed with the Primer Express 3.0 software. The cDNA samples were diluted, and mixed with gene-specific primers and the Fast SYBR Green Master Mix (Applied Biosystems, Foster City, CA) according to manufacture’s recommendations. The transcripts of the ribosome protein gene *rpl6e* were used for normalization of the qRT-PCR data, because this gene was shown to be a suitable reference for light/darkness transcription analysis in *T. reesei* [43]. The quantifications were performed by the Applied Biosystems 7500 fast Real-Time PCR system with default settings. The normalized expression levels of genes were analyzed with the 7500 Software V2.0.6.

### Microarray Experiments and Data Analysis

Microarray experiments were performed using the gene expression full service provided by the Microarray facility and the Bioinformatics Core in the Institute of Molecular Biology, Academia Sinica. Custom-designed oligonucleotide array by Roche-NimbleGen based on *Trichoderma reesei* v2.0 genome sequence using the 4×72000 formation [43]. Invitrogen SuperScript double-stranded cDNA synthesis kit was applied according to following the manufacturer’s instructions. Labeling and hybridization were performed by using NimbleGen Systems (NG_Expression_UGuide_v6p0). NimbleGen expression arrays were scanned with a GenePix 4000B Scanner and associated software Genepix6.0. All the microarray experiment were performed following the vendor’s standard operating protocol (NimbleGen Arrays User’s Guide: Gene Expression Analysis v3.0). Image data were processed using NimbleScan software version 2.6.3 (Roche NimbleGen) to obtain the raw intensity data (.pair file) and converted to gene-leveled data(.calls). All experiments were done in technical triplicate with at least three different biological replicates. Data analysis and normalization were performed using Agilent Gene Spring GX 12.0. Raw intensity scales were transformed by quantile normalization, which used to correct array biases and to make all distributions the same. Both t-test and fold change criteria were employed simultaneously to identify differentially expressed genes with p-value < = 0.05 and fold-change > = 2. For the evaluation of results, the community annotation including the GO (Gene Ontology) classification is available at the *T. reesei* genome database v2.0 (http://genome.jgi-psf.org/Trire2/Trire2.home.html).

## Supporting Information

Table S1 **193 genes were at least two-fold down-regulated (p value <0.05) under 4 sexually potent conditions (i.e., W-12L12D, W-12D12L, W-24D and E-24D) in comparison with those under the four sexually impotent conditions (i.e., E-12D12L, E-12L12D, E-24L and W-24L).**
(XLS)

Table S2 **287 genes are at least two-fold up-regulated (p value <0.05) under 4 sexually potent conditions (i.e., W-12L12D, W-12D12L, W-24D and E-24D) in comparison with those under the three sexually impotent conditions (i.e., E-12D12L, E-12L12D, E-24D).**
(XLS)

Table S3 **Nucleotide sequence of primers used for preparing DNA probes in Northern blot analysis and for qRT-PCR analysis.**
(DOC)
