# Supplementary material for: Blue Light Acts as a Double-Edged Sword in Regulating Sexual Development of Hypocrea jecorina (Trichoderma reesei)
Source: PLoS One. 2012 Sep 18;7(9):e44969. doi: 10.1371/journal.pone.0044969 (PMC3445611; doi:10.1371/journal.pone.0044969)
Supplement: Table S3 — Nucleotide sequence of primers used for preparing DNA probes in Northern blot analysis and for qRT-PCR analysis. (DOC) [file pone.0044969.s003.doc]

Table S3. Primers

| Primer | Name | Nucleotide Sequence |
| --- | --- | --- |
| 1. Northern blot |  |  |
| actin-R | PA6693 | 5’-ATGATCGGTATGGGTCAG -3’ |
| actin-F | PA6694 | 5’-GATGTCACGGACGATTTC-3’ |
| env1-F | PA6185 | 5’-CCTTGATGAAATCTCGAGTG-3’ |
| env1-R | PA6150 | 5’-TGCCTAGTTACGAATTGCTT-3’ |
| 2. qRT-PCR |  |  |
| rpl6E-R | PA6776 | 5’-TGACAAGCCCCACGAGATG-3’ |
| rpl6E-F | PA6777 | 5’-GCCTACCCGAGATTTTGTGTGT-3’ |
| hpp1-F | PA6651 | 5’-CAGCTCTTCAAAAACCACATCAA-3’ |
| hpp1-R | PA6652 | 5’-CAGCCAATGAGCCTCTTCCT-3’ |
| env1-F | PA6675 | 5’-CGAATCCGACCATTGACCTT-3’ |
| env1-R | PA6676 | 5’-CGGTAAGCTGATATCGCACAAG-3’ |
| con6-F | PA6744 | 5’-GGAACCGAGACTAAGCAAAAGG-3’ |
| con6-R | PA6745 | 5’-CTCCGACACGCTCTCATTTGT-3’ |
| con10-F | PA6746 | 5’-CAAGGAGGAGGTTCAAAGCATT-3’ |
| con10 R | PA6747 | 5’-ACCCTTGGATGCGATTTCC-3’ |
| Trire2_57101-F | PA6780 | 5’-GCAGGTCCTCGGCATCTTC-3’ |
| Trire2_57101-R | PA6781 | 5’-GCTGATTTCCGAGTAGAGGTAGTAGAA-3’ |
| Trire2_30465-F | PA6782 | 5’-GGTCACGGGTTCCTGCAAT-3’ |
| Trire2_30465-R | PA6783 | 5’-GCCCAGGAGACAGCATTTACC-3’ |
| Trire2_107856-F | PA6786 | 5’-AGCTTTGGCTTGCGAATCA-3’ |
| Trire2_107856-R | PA6787 | 5’-TTGTGCCGAGAAGATGAAGGA-3’ |
| Trire2_121135-F | PA6790 | 5’-AAAGGACTCCTACTACACACTGACGTT-3’ |
| Trire2_121135-R | PA6791 | 5’-GCCCAAGCCACGACAGAT-3’ |
